# High Prevalence of Anti-PF4 Antibodies Following ChAdOx1 nCov-19 (AZD1222) Vaccination Even in the Absence of Thrombotic Events

**DOI:** 10.3390/vaccines9070712

**Published:** 2021-07-01

**Authors:** Evangelos Terpos, Marianna Politou, Ioannis Ntanasis-Stathopoulos, Vangelis Karalis, Efrosyni Merkouri, Despina Fotiou, Maria Gavriatopoulou, Panagiotis Malandrakis, Efstathios Kastritis, Ioannis P. Trougakos, Meletios A. Dimopoulos

**Affiliations:** 1 Department of Clinical Therapeutics, School of Medicine, National and Kapodistrian University of Athens, PS 11528 Athens, Greece; johnntanasis@med.uoa.gr (I.N.-S.); desfotiou@med.uoa.gr (D.F.); mariagabria@gmail.com (M.G.); panosmalandrakis@gmail.com (P.M.); ekastritis@gmail.com (E.K.); mdimop@med.uoa.gr (M.A.D.); 2 Hematology Laboratory-Blood Bank, Aretaieio Hospital, School of Medicine, National and Kapodistrian University of Athens, PS 11528 Athens, Greece; mpolitou@med.uoa.gr (M.P.); fmerk62@yahoo.gr (E.M.); 3 Department of Pharmacy, School of Health Sciences, National and Kapodistrian University of Athens, PS 15784 Athens, Greece; vkaralis@pharm.uoa.gr; 4 Department of Cell Biology and Biophysics, Faculty of Biology, National and Kapodistrian University of Athens, PS 15784 Athens, Greece; itrougakos@biol.uoa.gr

**Keywords:** SARS-CoV-2, COVID-19, ChAdOx1 nCov-19, anti-PF4, antibodies, vaccine, thrombosis

## Abstract

It is unclear whether the ChAdOx1 nCov-19 vaccine can induce the development of anti-PF4 antibodies in vaccinated individuals who have not developed thrombosis. The aim of this prospective study was to evaluate the presence of antibodies against heparin/PF4 in adults who received a first dose of the ChAdOx1 nCov-19 vaccine, and correlate them with clinical data and antibody responses to the vaccine. We detected non-platelet activating anti-PF4 antibodies in 67% (29/43) of the vaccinated individuals on day 22 following the first dose of the ChAdOx1 nCov-19 vaccine, though these were detected in low titers. Furthermore, there was no correlation between the presence of anti-PF4 IgG antibodies and the baseline clinical characteristics of the patients. Our findings suggest that the ChAdOx1 nCov-19 vaccine can elicit anti-PF4 antibody production even in recipients without a clinical manifestation of thrombosis. The presence of anti-PF4 antibodies was not sufficient to provoke clinically evident thrombosis. Our results offer an important insight into the ongoing investigations regarding the underlying multifactorial pathophysiology of thrombotic events induced by the ChAdOx1 nCov-19 vaccine.

## 1. Introduction

Severe Acute Respiratory Syndrome Coronavirus 2 (SARS-CoV-2), the causative agent of Coronavirus disease 2019 (COVID-19), has caused almost 3.2 M deaths worldwide. It affects not only the respiratory tract but almost all body systems and organs, including the blood, the heart, the endocrine glands, the central nervous system, etc. [1,2,3,4]. Vaccination programs against SARS-CoV-2 are progressing across the globe. The BNT162b2 mRNA and the ChAdOx1 nCov-19 (AZD1222) adenovirus vector vaccines account for the majority of vaccinations around the world following encouraging clinical trial results [5,6].

It has been recently reported that ChAdOx1 nCov-19, when used against SARS-CoV-2, can result in vaccine-induced immune thrombotic thrombocytopenia (VITT) [7]. VITT is a rare syndrome that clinically mimics autoimmune heparin-induced thrombocytopenia (HIT) [8,9]. Similar to HIT, special therapeutic treatment is indicated in the case of VITT, including the administration of non-heparin, non-APTT-adjusted anticoagulants, intravenous immunoglobulin, and possibly Bruton’s tyrosine kinase inhibitors [10,11,12]. In the majority of patients with VITT and thrombosis in uncommon sites, the diagnostic work-up has confirmed the presence of antibodies against heparin/PF4, although none of the patients had a history of exposure to heparin [7,13]. On the basis of these reports, it has been suggested that the diagnosis of VITT should be confirmed with an approved PF4 ELISA [13]. In addition, anti-PF4–related non-platelet activating antibodies have been detected in patients with COVID-19 who were suspected of having HIT [14,15].

It has yet to be elucidated whether anti-PF4 antibodies are a component of a broader immune complex that activates platelets, or whether they contribute directly to clot formation [16]. Furthermore, it is unclear whether the vaccine can induce the development of anti-PF4 antibodies (seroconversion) in vaccinated individuals who have not developed thrombosis [17], since the data published so far are limited to patients with overt thrombotic complications. Determining the indications of anti-PF4 antibody testing in vaccine recipients is essential for assuring best clinical practice and for avoiding misinformation [17,18]. In this context, the aim of this prospective study was to assess the presence of antibodies against heparin/PF4 in healthy adults who received the first dose of the ChAdOx1 nCov-19 vaccine, according to the national vaccination program, and correlate them with clinical data and antibody responses to the vaccine. Antibodies against PF4 were evaluated 22 days after the first dose of the vaccine, based on the known kinetics of adaptive immunity, both after COVID-19 natural infection and after vaccination [19,20,21].

## 2. Materials and Methods

### 2.1. Study Design

All individuals participated in a prospective study (NCT04743388) regarding the efficacy of vaccination for the prevention of COVID-19 in Greece. Here, we report the presence of antibodies against heparin/PF4 in adults who received the first dose of the ChAdOx1 nCov-19 vaccine in vaccination centers in Athens, Greece, and their correlation with clinical characteristics of vaccinated individuals and the development of antibodies against SARS-CoV-2 post-vaccination. Major inclusion criteria for participation in this study included: (i) age above 18 years; (ii) ability to sign the informed consent form; and (iii) eligibility for vaccination according to the national program for COVID-19 vaccination. Major exclusion criteria included the presence of: (i) an autoimmune disorder under immunosuppressive therapy and (ii) end-stage renal disease.

Data of the subjects were kept confidential in accordance with the General Data Protection Regulation (GDPR) rules (Regulation 2016/679 of European Parliament 2016). All names were kept confidential and, immediately after collection, names were deleted and randomly replaced with a unique number. The study was approved by the respective Ethical Committee of Alexandra Hospital (Ref No. 15/23.12.2020), in accordance with the Declaration of Helsinki and International Conference for harmonization for good clinical practice. All participants gave their written consent before their entry into the study.

### 2.2. Laboratory Methodology

Serum samples were collected and analyzed on the day of vaccination with the first shot of the ChAdOx1 nCov-19 vaccine (day 1; before the vaccine shot), as well as three weeks later (day 22). The samples collected on day 1 served as our control group. Following vein puncture, serum was separated within 4 h from blood collection and stored at −80 °C until we performed the assays. Samples in different time points from the same donor were measured for all individuals in parallel.

The presence of IgG antibodies against PF4/heparin was evaluated with an enzyme-linked immunosorbent assay (ELISA, Asserachrom, Stago, Vienna, Austria). All absorbance values greater than the ×11% of the absorbance value observed for the control were considered positive, as was instructed by the manufacturer.

The ability of participants’ serum, which was positive for anti PF4 antibodies, to aggregate platelets in the presence of unfractionated heparin (UFH) was also tested with whole blood impedance aggregometry on a Multiplate Platelet Analyzer (Roche Diagnostics, Switzerland) [22]. Briefly, whole blood from a healthy donor, as a source of non-activated platelets, was mixed with heparin in three different concentrations (a high concentration solution of 400 U/mL, an intermediate concentration solution of 100 U/mL, and a low concentration solution of 2 U/mL) and a 0.9% saline solution as negative control. The samples were incubated for 6 min in the multiplate measuring cells. After the incubation, 200 μL of the participant’s plasma was added in order to trigger platelet aggregation. We observed eventual activation during 20 min. Each run included a known positive control of a non-vaccinated patient with no history of COVID-19 who had tested positive for HIT and a negative control from a healthy donor who tested negative for PF4 antibodies. According to the manufacturer’s instructions, the tests were always performed in duplicate and a coefficient of variation was applied before software calculation. Duplicates with a difference superior to 20% were repeated. The donor’s platelets efficiency to aggregate was tested by using a parallel run with Thrombin Receptor Activating Peptide (TRAP) as an activator.

Finally, we also evaluated the humoral response to the ChAdOx1 nCov-19 vaccine by measuring neutralizing antibodies (NAbs) against SARS-CoV-2 on days 1 and 22. We used an FDA-approved methodology, as previously described [23,24]. In summary, the cPass™ SARS-CoV2 NAbs Detection Kit (GenScript, Piscataway, NJ, USA) was applied, which allowed for the indirect detection of potential SARS-CoV-2 NAbs in the blood by testing the antibody-mediated inhibition of SARS-CoV-2 RBD binding to the human host receptor angiotensin converting enzyme-2. Based on the assay, neutralizing titers were considered positive if the value was above 30%, while clinically significant neutralizing titers were those equal to or above 50% [25]; the FDA has defined as high titer for this methodology as any value above 68%.

### 2.3. Statistical Analysis

Descriptive statistics were first applied to summarize the data, describe the characteristics of the participants, and obtain estimates for the median/range of anti-PF4 levels and neutralizing antibodies to SARS-Cov-2. Before statistical comparisons, normality assessment of variables was performed to choose between parametric and non-parametric methods. Shapiro–Wilk and QQ plots were used to assess the normality of the data distribution.

For all analyses in this study, the significance level was set at 5%. In the case of comparisons, a *p*-value of less than 5% indicated a statistically significant difference while, for the Shapiro–Wilk test, a *p*-value of less than 5% implied a rejection of the nominal hypothesis and the conclusion that the data did not follow a normal distribution. In this analysis, none of the variables were found to follow a normal distribution; therefore, non-parametric methods were used.

Anti-PF4 antibody levels were compared between day 1 (before vaccine injection) and day 22 (three weeks after vaccination). Since the comparison involved the same individuals, the Wilcoxon signed-rank test was used, as it is the appropriate non-parametric method for comparing paired groups. Correlations between anti-PF4 antibodies and neutralizing antibodies were assessed using scatter plots and by estimating Pearson’s and Spearman’s coefficients. All statistical analysis was performed in the Python programming language (v. 3.9.2, Python Software Foundation, Wilmington, DE, USA).

## 3. Results

### 3.1. Patient Characteristics

Forty-three individuals who were vaccinated with a first dose of the ChAdOx1 nCov-19 vaccine between 20 February 2021 and 31 March 2021 in Athens (Greece) participated in the study (Table 1).

According to the Greek National Immunization Program, only individuals aged 60–64 years had access to the AZ vaccine during the study period; thus, all participants belonged to this age group. The median age of the study participants was 62 years (range 60–64 years), while 22 participants were male and 21 were female. Thirty individuals were health care workers and thirteen were patients who had hematological malignancies (multiple myeloma or Waldenström’s macroglobulinemia), and they were followed-up in the Plasma Cell Dyscrasias Unit of the National and Kapodistrian University of Athens, School of Medicine in Alexandra General Hospital of Athens (Greece).

Regarding comorbidities, 14 (32.5%) participants had hyperlipidemia under treatment with statins, 12 (28%) had hypertension, 11 (25.5%) had diabetes mellitus, 4 (9.3%) had hypothyroidism due to Hashimoto disease under T4 therapy, and 2 (4.6%) had gastroesophageal reflux disease under treatment with proton pump inhibitors (Table 1). We also evaluated the body mass index (BMI) of the participants: 12 (27.9%) subjects had normal weight, 18 (41.8%) were overweight, and 13 (30.2%) were obese. No individual had chronic cardiac disease or any other cardiac disorder that required therapy. Furthermore, none of the study participants had a known history of exposure to heparin or any other anti-coagulant.

The first vaccine dose was well tolerated. Sixteen (37.2%) participants experienced no adverse events after vaccination, while 27 (62.7%) experienced at least one grade 1/2 adverse event, including fever, chills, fatigue, myalgia, or pain at the injection site that lasted from 24 h to 6 days (in two individuals). No severe adverse event of grade 3 or more was observed with the first vaccine dose.

### 3.2. Measurement of PF4/Heparin IgG Antibodies

**Figure 1:**
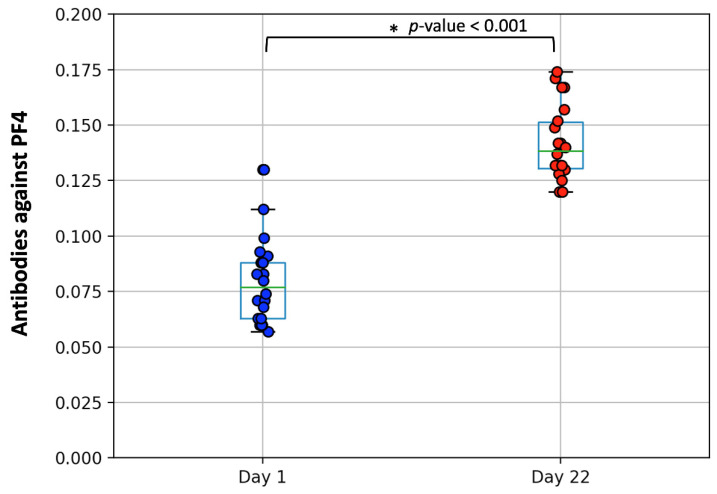
Distribution of anti-PF4/heparin antibody values on the day of vaccination with the first shot of ChAdOx1 nCov-19 vaccine (day 1, median 0.078, range 0.073) and after 3 weeks (day 22, median 0.139, range 0.054). The boxplot borders refer to the 25th and 75th quartiles of the distribution, while the line inside is the median. The asterisk (*) denotes the *p*-value (<0.001) for the comparison of anti-PF4 levels between the two days according to the Wilcoxon signed ranked test.

We found that none of the recipients had detectable PF4/heparin IgG antibodies on the day of vaccine administration (median 0.078, range 0.073) (control group). However, antibodies were detected in 67% (29/43) of the study participants on day 22, though in low titers; namely, low anti-PF4 antibodies were recorded, (median 0.139, range 0.054) (Figure 1, *p*-value < 0.001 for the Wilcoxon signed ranked test). The positivity rate among health workers was 64% (20/31) and 75% among patients (9/12) with hematologic malignancies. Among the latter, the administration of chemotherapy was not associated with the presence of anti-PF4 antibodies.

Furthermore, we evaluated the ELISA-positive serum samples (*n* = 29) for their ability to aggregate platelets in the presence of unfractionated heparin (UFH), as described in the Methods session. We found that, although the samples tested were positive for anti-PF4 antibodies, none of them showed a functional ability to provoke an inappropriate activation of the donor’s platelets with any concentration of heparin added, thus excluding the presence of HIT.

### 3.3. Correlations with Antibody Response and Clinical Characteristics

**Figure 2:**
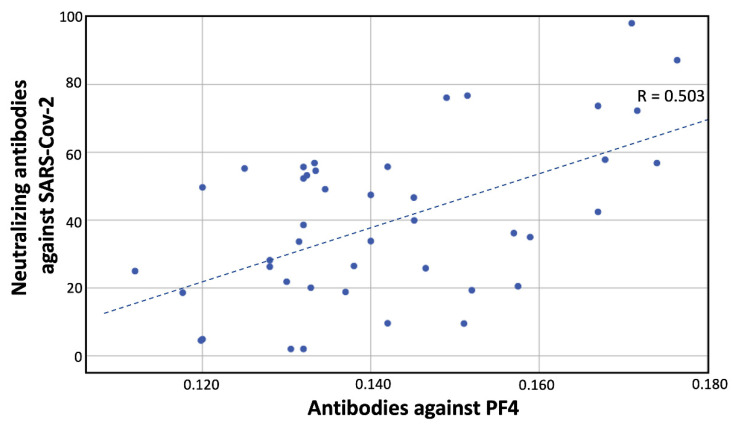
Correlation between neutralizing antibodies against SARS-CoV-2 and antibodies against PF4 at day 22 (R = 0.503 refers to the correlation coefficient and *p* value is 0.017). Dashed line corresponds to the regression line describing the pairs (anti-PF4 antibodies and neutralizing antibodies) of data.

Interestingly, the titer of the IgG antibodies against PF4 correlated linearly (correlation coefficient equal to 0.503, *p*-value = 0.017) with the titre of neutralizing antibodies against SARS-CoV-2 (Figure 2).

There was no correlation of the presence of anti-PF4 IgG antibodies with clinical characteristics of the patients, i.e., specific comorbidities, BMI, or the development of side-effects after the first vaccine dose.

## 4. Discussion

Our findings suggest that the ChAdOx1 nCov-19 vaccine can elicit anti-PF4 antibody production, even in recipients without a clinical manifestation of thrombosis. In accordance with our results, Thiele et al. have recently reported that up to 6.8% of the healthy individuals vaccinated with anti-SARS-CoV-2 vaccines may present anti-PF4 antibodies following vaccination [26]. More specifically, the frequency of anti-PF4 antibodies was 5.6% (95% CI: 2.9–10.7%) with BNT162b2 and 8.0% (95% CI: 4.5–13.7%) with ChAdOx1 nCoV-19 [26]. It has to be noted that the optical densities were mainly low (range 0.5–1.0, positivity cut-off 0.5) among the participants with anti-Pf4/polyanion antibodies, whereas the addition of PF4 did not induce platelet activation in any of the positive sera [26]. In another study including health care workers vaccinated with ChAdOx1 nCov-19, an even lower incidence of anti-PF4 antibodies was reported at 1.4% [27]. Similar to the previous study, the optical densities of the positive sera were low, ranging from 0.58 to 1.16 (positivity cut-off 0.4). Importantly, the presence of anti-PF4/polyanion antibodies was not correlated with reduced platelet counts [27]. The differences in the percentages between the studies may be attributed to the variable sensitivities of the laboratory assays used [28]. Low titers of anti-PF4 antibodies have been detected following both BNT162b2 and ChAdOx1 nCov-19 vaccination and, more importantly, these antibodies do not activate platelets in the presence of PF4 [26]. Therefore, the clinical relevance of anti-PF4 antibodies in vaccinated persons is rather limited.

Our results may provide an indirect implication that anti-PF4 antibodies alone cannot be the driver event for the development of VITT in recipients of the ChAdOx1 nCov-19 vaccine. However, they could contribute as a part of an extended immune response, leading to a hypercoagulable state (“immunothrombosis”) with thrombin generation and platelet consumption [12,17]. An excess inflammatory response, probably due to the adenoviral vector component of the ChAdOx1 nCoV-19, results in the release of PF4 contained in the platelets. The subsequent development of autoreactive antibodies against PF4 may drive the activation of immune cells and subsequent thrombin generation and platelet consumption [12]. Thrombin production may be also triggered by the increased levels of tissue factor due to vascular inflammation [12,29]. Furthermore, a hyper-inflammatory state may lead to the release of neutrophil extracellular traps (NETs) consisting of leukocytic DNA that favor microthrombi formation [30,31,32]. CD32a is considered a key molecule in the immune cascade of platelet hyperactivation that is mediated by autoreactive anti-PF4 antibodies, whereas the administration of polyclonal immunoglobulin in high concentrations is able to counteract this phenomenon [8,13,33].

It has been demonstrated that antibodies directed against HPF4 complexes are produced in approximately 85% of HIT cases. They are mainly of the IgG class but may also be of either the IgA or IgM class; however, IgM and IgA anti-PF4 antibodies, as well as non-PF4-dependent antigens, are not usually implicated in HIT [34]. We have to keep in mind that results obtained with HPF4 measurement must be interpreted in relation to the function of both the clinical state of the patient and in terms of platelet kinetics. The presence of anti-PF4 antibodies is not sufficient to provoke thrombosis. ELISA-based diagnostics are highly sensitive (up to 99%) but they have relatively low specificity, which may lead to overdiagnosis of HIT syndrome [34,35,36]. Indeed, about 50% of the patients with clinical suspicion of HIT and positive ELISA demonstrate a positive platelet activation assay [34,35]. A high prevalence of PF4/heparin antibodies has been reported in cardiosurgical and orthopedic patients, as well as in volunteer blood donors without a clinical syndrome of thrombosis/thrombocytopenia [29,35,37,38,39]. Among approximately 4000 blood bank donors, antibodies against PF4/heparin by ELISA were detected in 249 individuals (6.6%) [37]. However, a confirmatory evaluation was positive in 163 (4.3%). The vast majority of false positives had marginal values of optical density, ranging from 0.40 to 0.59. Similar values were also reported for the 71% of the confirmed positive samples. Heparin-dependent binding of the detected anti-PF4 antibodies was demonstrated in 124 (76%) of the 163 confirmed positive samples [37]. Interestingly, Krauel et al. demonstrated that PF4 binds to bacteria during infection and induces the production of antibodies against PF4/polyanion complexes that enhance antibody-dependent bacterial phagocytosis [40]. Therefore, the immune system of some individuals may be primed to recognize PF4/heparin due to preimmunization by bacterial antigens with similar structures [40].

Taking into consideration all the above, the diagnosis of VITT syndrome following vaccination with the ChAdOx1 nCov-19 vaccine cannot be solely attributable to the presence of anti-PF4 antibodies detected by ELISA assays. A list of clinical signs and symptoms can be used in order to raise suspicion of VITT syndrome and guide the diagnostic algorithm. These “red flags” typically appear between 4 and 28 days post vaccination, and include loss of consciousness, double and/or blurred vision, sudden loss of balance with accompanying dizziness and/or severe headache, severe and persistent abdominal pain, nausea, diarrhea, vomiting, bloody or tarry stools, shortness of breath with or without chest pain, and edema of an upper or lower extremity [17]. It should be noted that VITT is a rare complication and the anticipated benefits of anti-SARS-CoV-2 vaccination outweigh the possible risks. The still accumulating knowledge on the underlying pathophysiology of this syndrome and experience in the management of patients will contribute to the optimization of a diagnostic and therapeutic approach in order to prevent functional impairment and life loss due to VITT. The education of both health care personnel, with specialized algorithms for differential diagnosis and therapeutics, and the general population in simplified language is essential for increasing confidence in the indispensable vaccination effort against SARS-CoV-2.

Our study has some limitations that have to be considered during the interpretation and the generalization of our findings. Although we used the baseline values of anti-PF4 antibodies of the participants as an internal control group, we did not include an independent control group of non-vaccinated healthy individuals with no history of prior COVID-19 as an external control group. Similarly, we did not include an external control group consisting of individuals vaccinated with other vaccine types. Furthermore, an investigation into a larger number of vaccinated individuals may be necessary to perform subgroup analyses in order to evaluate the possible role of confounding factors in the production of anti-PF4 antibodies and the presence of thrombosis following vaccination with the ChAdOx1 nCov-19 vaccine. In addition to the above, the evaluation of platelet activation by a more sensitive and specific functional assay, such as the serotonin release assay, would have enhanced our findings further.

Last but not least, a further investigation of the pathophysiology in an in vivo model is needed. Studying the presence of PF4 antibodies in recipients of other types of vaccines and after natural infection could help us characterize the triggering antigens (adenovirus, spike protein, or other) that induce the production of such antibodies. These data would be valuable in improving the design of safe, next-generation vaccines against SARS-CoV-2.

## 5. Conclusions

Our findings suggest that the ChAdOx1 nCov-19 vaccine can elicit anti-PF4 antibody production even in recipients without a clinical manifestation of thrombosis. The presence of anti-PF4 antibodies was not sufficient to provoke clinically evident thrombosis. Our results offer an important insight into the ongoing investigations regarding the underlying multifactorial pathophysiology of thrombotic events induced by the ChAdOx1 nCov-19 vaccine.

## Figures and Tables

**Table 1:** Characteristics of the participants in the study.

Characteristic	Value
Number of participants	43
Age (median, range) (years)	62 (60–64)
Gender (*n*, %)	
Women	21 (48.8%)
Men	22 (51.2%)
BMI (*n*, %)	
Normal weight	12 (27.9%)
Overweight	18 (41.8%)
Obese	13 (30.2%)
Co-morbidities of all participants (*n*, %)	
Hyperlipidemia	14 (32.5%)
Hypertension	12 (28%)
Diabetes mellitus	11 (25.5%)
Hypothyroidism	4 (9.3%)
Gastroesophageal reflux disease	2 (4.6%)
Hematologic malignancy Current treatment	12 (28%) 6/12 (50%)
Co-morbidities of women (*n*, %)	
Hyperlipidemia	6 (13.9%)
Hypertension	2 (4.65%)
Diabetes mellitus	7 (16.3%)
Hypothyroidism	3 (7.0%)
Gastroesophageal reflux disease	1 (2.3%)

## Data Availability

Raw data are available upon request from the corresponding author.

## References

[1] Gavriatopoulou M., Korompoki E., Fotiou D., Ntanasis-Stathopoulos I., Psaltopoulou T., Kastritis E., Terpos E., Dimopoulos M.A. (2020). Organ-specific manifestations of COVID-19 infection. Clin. Exp. Med..

[2] Trougakos I.P., Stamatelopoulos K., Terpos E., Tsitsilonis O.E., Aivalioti E., Paraskevis D., Kastritis E., Pavlakis G.N., Dimopoulos M.A. (2021). Insights to SARS-CoV-2 life cycle, pathophysiology, and rationalized treatments that target COVID-19 clinical complications. J. Biomed. Sci..

[3] Gavriatopoulou M., Ntanasis-Stathopoulos I., Korompoki E., Fotiou D., Migkou M., Tzanninis I.G., Psaltopoulou T., Kastritis E., Terpos E., Dimopoulos M.A. (2021). Emerging treatment strategies for COVID-19 infection. Clin. Exp. Med..

[4] Korompoki E., Gavriatopoulou M., Hicklen R.S., Ntanasis-Stathopoulos I., Kastritis E., Fotiou D., Stamatelopoulos K., Terpos E., Kotanidou A., Hagberg C.A. (2021). Epidemiology and organ specific sequelae of post-acute COVID19: A Narrative Review. J. Infect..

[5] Polack F.P., Thomas S.J., Kitchin N., Absalon J., Gurtman A., Lockhart S., Perez J.L., Perez Marc G., Moreira E.D., Zerbini C. (2020). Safety and Efficacy of the BNT162b2 mRNA Covid-19 Vaccine. N. Engl. J. Med..

[6] Ramasamy M.N., Minassian A.M., Ewer K.J., Flaxman A.L., Folegatti P.M., Owens D.R., Voysey M., Aley P.K., Angus B., Babbage G. (2021). Safety and immunogenicity of ChAdOx1 nCoV-19 vaccine administered in a prime-boost regimen in young and old adults (COV002): A single-blind, randomised, controlled, phase 2/3 trial. Lancet.

[7] Greinacher A., Thiele T., Warkentin T.E., Weisser K., Kyrle P.A., Eichinger S. (2021). Thrombotic Thrombocytopenia after ChAdOx1 nCov-19 Vaccination. N. Engl. J. Med..

[8] Schultz N.H., Sorvoll I.H., Michelsen A.E., Munthe L.A., Lund-Johansen F., Ahlen M.T., Wiedmann M., Aamodt A.H., Skattor T.H., Tjonnfjord G.E. (2021). Thrombosis and Thrombocytopenia after ChAdOx1 nCoV-19 Vaccination. N. Engl. J. Med..

[9] Franchini M., Liumbruno G.M., Pezzo M. (2021). COVID-19 Vaccine-associated Immune Thrombosis and Thrombocytopenia (VITT): Diagnostic and therapeutic recommendations for a new syndrome. Eur. J. Haematol..

[10] Greinacher A., Selleng K., Warkentin T.E. (2017). Autoimmune heparin-induced thrombocytopenia. J. Thromb. Haemost..

[11] Warkentin T.E. (2019). High-dose intravenous immunoglobulin for the treatment and prevention of heparin-induced thrombocytopenia: A review. Expert Rev. Hematol..

[12] Von Hundelshausen P., Lorenz R., Siess W., Weber C. (2021). Vaccine-induced immune thrombotic thrombocytopenia (VITT): Targeting pathomechanisms with Bruton tyrosine kinase inhibitors. Thromb. Haemost..

[13] Scully M., Singh D., Lown R., Poles A., Solomon T., Levi M., Goldblatt D., Kotoucek P., Thomas W., Lester W. (2021). Pathologic Antibodies to Platelet Factor 4 after ChAdOx1 nCoV-19 Vaccination. N. Engl. J. Med..

[14] Nazy I., Jevtic S.D., Moore J.C., Huynh A., Smith J.W., Kelton J.G., Arnold D.M. (2021). Platelet-activating immune complexes identified in critically ill COVID-19 patients suspected of heparin-induced thrombocytopenia. J. Thromb. Haemost..

[15] Brodard J., Kremer Hovinga J.A., Fontana P., Studt J.D., Gruel Y., Greinacher A. (2021). COVID-19 patients often show high-titer non-platelet-activating anti-PF4/heparin IgG antibodies. J. Thromb. Haemost..

[16] Kadkhoda K. (2021). Post-adenoviral-based COVID-19 vaccines thrombosis: A proposed mechanism. J. Thromb. Haemost..

[17] Elalamy I., Gerotziafas G., Alamowitch S., Laroche J.P., van Dreden P., Ageno W., Beyer-Westendorf J., Cohen A.T., Jimenez D., Brenner B. (2021). SARS-CoV-2 vaccine and thrombosis: Expert opinions. Thromb. Haemost..

[18] Tentolouris A., Ntanasis-Stathopoulos I., Vlachakis P.K., Tsilimigras D.I., Gavriatopoulou M., Dimopoulos M.A. (2021). COVID-19: Time to flatten the infodemic curve. Clin. Exp. Med..

[19] Grigoryan L., Pulendran B. (2020). The immunology of SARS-CoV-2 infections and vaccines. Semin. Immunol..

[20] Reynolds C.J., Pade C., Gibbons J.M., Butler D.K., Otter A.D., Menacho K., Fontana M., Smit A., Sackville-West J.E., Cutino-Moguel T. (2021). Prior SARS-CoV-2 infection rescues B and T cell responses to variants after first vaccine dose. Science.

[21] Post N., Eddy D., Huntley C., van Schalkwyk M.C.I., Shrotri M., Leeman D., Rigby S., Williams S.V., Bermingham W.H., Kellam P. (2020). Antibody response to SARS-CoV-2 infection in humans: A systematic review. PLoS ONE.

[22] Morel-Kopp M.C., Mullier F., Gkalea V., Bakchoul T., Minet V., Elalamy I., Ward C.M. (2016). Subcommittee on Platelet Immunology; Heparin-induced multi-electrode aggregometry method for heparin-induced thrombocytopenia testing: Communication from the SSC of the ISTH. J. Thromb. Haemost..

[23] Terpos E., Trougakos I.P., Gavriatopoulou M., Papassotiriou I., Sklirou A.D., Ntanasis-Stathopoulos I., Papanagnou E.D., Fotiou D., Kastritis E., Dimopoulos M.A. (2021). Low Neutralizing Antibody Responses Against SARS-CoV-2 in Elderly Myeloma Patients After the First BNT162b2 Vaccine Dose. Blood.

[24] Terpos E., Trougakos I.P., Apostolakou F., Charitaki I., Sklirou A.D., Mavrianou N., Papanagnou E.D., Liacos C.I., Gumeni S., Rentziou G. (2021). Age-dependent and gender-dependent antibody responses against SARS-CoV-2 in health workers and octogenarians after vaccination with the BNT162b2 mRNA vaccine. Am. J. Hematol..

[25] Walsh E.E., Frenck R.W., Falsey A.R., Kitchin N., Absalon J., Gurtman A., Lockhart S., Neuzil K., Mulligan M.J., Bailey R. (2020). Safety and Immunogenicity of Two RNA-Based Covid-19 Vaccine Candidates. N. Engl. J. Med..

[26] Thiele T., Ulm L., Holtfreter S., Schonborn L., Kuhn S.O., Scheer C., Warkentin T.E., Broker B., Becker K., Aurich K. (2021). Frequency of positive anti-PF4/polyanion antibody tests after COVID-19 vaccination with ChAdOx1 nCoV-19 and BNT162b2. Blood.

[27] Sorvoll I.H., Horvei K.D., Ernstsen S.L., Laegreid I.J., Lund S., Gronli R.H., Olsen M.K., Jacobsen H.K., Eriksson A., Halstensen A.M. (2021). An observational study to identify the prevalence of thrombocytopenia and anti-PF4/polyanion antibodies in Norwegian health care workers after COVID-19 vaccination. J. Thromb. Haemost..

[28] Platton S., Bartlett A., MacCallum P., Makris M., McDonald V., Singh D., Scully M., Pavord S. (2021). Evaluation of laboratory assays for anti-Platelet Factor 4 antibodies after ChAdOx1 nCOV-19 vaccination. J. Thromb. Haemost..

[29] Warkentin T.E., Makris M., Jay R.M., Kelton J.G. (2008). A spontaneous prothrombotic disorder resembling heparin-induced thrombocytopenia. Am. J. Med..

[30] Merad M., Martin J.C. (2020). Pathological inflammation in patients with COVID-19: A key role for monocytes and macrophages. Nat. Rev. Immunol..

[31] McGonagle D., O’Donnell J.S., Sharif K., Emery P., Bridgewood C. (2020). Immune mechanisms of pulmonary intravascular coagulopathy in COVID-19 pneumonia. Lancet Rheumatol..

[32] Yu J., Yuan X., Chen H., Chaturvedi S., Braunstein E.M., Brodsky R.A. (2020). Direct activation of the alternative complement pathway by SARS-CoV-2 spike proteins is blocked by factor D inhibition. Blood.

[33] Padmanabhan A., Jones C.G., Pechauer S.M., Curtis B.R., Bougie D.W., Irani M.S., Bryant B.J., Alperin J.B., Deloughery T.G., Mulvey K.P. (2017). IVIg for Treatment of Severe Refractory Heparin-Induced Thrombocytopenia. Chest.

[34] Greinacher A., Juhl D., Strobel U., Wessel A., Lubenow N., Selleng K., Eichler P., Warkentin T.E. (2007). Heparin-induced thrombocytopenia: A prospective study on the incidence, platelet-activating capacity and clinical significance of antiplatelet factor 4/heparin antibodies of the IgG, IgM, and IgA classes. J. Thromb. Haemost..

[35] Juhl D., Eichler P., Lubenow N., Strobel U., Wessel A., Greinacher A. (2006). Incidence and clinical significance of anti-PF4/heparin antibodies of the IgG, IgM, and IgA class in 755 consecutive patient samples referred for diagnostic testing for heparin-induced thrombocytopenia. Eur. J. Haematol..

[36] Warkentin T.E. (2019). Laboratory diagnosis of heparin-induced thrombocytopenia. Int. J. Lab. Hematol..

[37] Hursting M.J., Pai P.J., McCracken J.E., Hwang F., Suvarna S., Lokhnygina Y., Bandarenko N., Arepally G.M. (2010). Platelet factor 4/heparin antibodies in blood bank donors. Am. J. Clin. Pathol..

[38] Pruthi R.K., Daniels P.R., Nambudiri G.S., Warkentin T.E. (2009). Heparin-induced thrombocytopenia (HIT) during postoperative warfarin thromboprophylaxis: A second example of postorthopedic surgery ‘spontaneous’ HIT. J. Thromb. Haemost..

[39] Jay R.M., Warkentin T.E. (2008). Fatal heparin-induced thrombocytopenia (HIT) during warfarin thromboprophylaxis following orthopedic surgery: Another example of ‘spontaneous’ HIT?. J. Thromb. Haemost..

[40] Krauel K., Potschke C., Weber C., Kessler W., Furll B., Ittermann T., Maier S., Hammerschmidt S., Broker B.M., Greinacher A. (2011). Platelet factor 4 binds to bacteria, [corrected] inducing antibodies cross-reacting with the major antigen in heparin-induced thrombocytopenia. Blood.

